# A new model to predict acute kidney injury after cardiac surgery in patients with renal insufficiency

**DOI:** 10.1080/0886022X.2022.2071297

**Published:** 2022-05-03

**Authors:** Xijian Wang, Naifeng Guo, Ying Chen, Houyong Dai

**Affiliations:** a Department of Nephrology, Affiliated Hospital of Nantong University, Nantong Jiangsu, China; b Department of Epidemiology and Medical Statistics, Nantong University School of Public Health, Nantong Jiangsu, China

**Keywords:** Acute kidney injury, renal replacement therapy, renal insufficiency, cardiac surgery, risk model

## Abstract

**Objective:**

To establish a simple model for predicting postoperative acute kidney injury (AKI) requiring renal replacement therapy (RRT) in patients with renal insufficiency (CKD stages 3–4) who underwent cardiac surgery.

**Methods:**

A total of 330 patients were enrolled. Among them, 226 were randomly selected for the development group and the remaining 104 for the validation group. The primary outcome was AKI requiring RRT. A nomogram was constructed based on the multivariate analysis with variables selected by the application of the least absolute shrinkage and selection operator. Meanwhile, the discrimination, calibration, and clinical power of the new model were assessed and compared with those of the Cleveland Clinic score and Simplified Renal Index (SRI) score in the validation group. Results: The rate of RRT in the development group was 10.6% (*n* = 24), while the rate in the validation group was 14.4% (*n* = 15). The new model included four variables such as postoperative creatinine, aortic cross‐clamping time, emergency, and preoperative cystatin C, with a C-index of 0.851 (95% CI, 0.779–0.924). In the validation group, the areas under the receiver operating characteristic curves for the new model, SRI score, and Cleveland Clinic score were 0.813, 0.791, and 0.786, respectively. Furthermore, the new model demonstrated greater clinical net benefits compared with the Cleveland Clinic score or SRI score.

**Conclusions:**

We developed and validated a powerful predictive model for predicting severe AKI after cardiac surgery in patients with renal insufficiency, which would be helpful to assess the risk for severe AKI requiring RRT.

## Introduction

With the rapid growth of the aging population and the increasing number of chronic diseases, such as hypertension, arteriosclerosis, and diabetes, more and more patients with renal insufficiency will be referred to cardiac surgery [1–4]. Whereas, the reduction of renal function reserve as a result of underlying chronic kidney disease (CKD) makes the kidney more vulnerable to this operation. The incidence of acute kidney injury (AKI) after cardiac surgery varies from 7% to 40%, and about 1% of them need renal replacement therapy (RRT) [5–7]. Obviously, the prevalence will be more serious in renal insufficiency patients, especially in patients with CKD stages 3–4 due to the lack of renal function reserve. The development of AKI requiring RRT is associated with worse postoperative outcomes and higher costs in patients undergoing cardiac surgery [8]. Therefore, given the importance to patients and cost implications of RRT, there is an urgent need to predict the occurrence of AKI, especially in patients with renal insufficiency.

Several models for cardiac surgery patients to predict severe AKI requiring RRT have been established, including the Cleveland Clinic score and the Simplified Renal Index (SRI) score [9,10]. However, there are some limitations to their adoption, including the measurement of RRT risk, time consumption and complexity of variables required, and poor discrimination among patients at the highest risk. The pathophysiology of cardiac surgery-associated AKI appears to be complex because of various risk factors, resulting in significant heterogeneity among different results [11]. Patients with renal insufficiency often have different comorbidities and risk factors for AKI than general patients [1].

Ideally, as a risk prediction model for clinical application, it should have a better clinical utility and reliability, and incorporate as few variables as possible to make it rapidly accessible for the clinician. To our knowledge, there are no previous retrospective studies that develop the RRT risk model after cardiac surgery in patients with renal insufficiency. In this study, the preoperative, intraoperative, and immediate postoperative data were collected, which could be helpful to improve the model discrimination while reducing the number of variables.

The aim of the present study was to establish a simple risk model to enable bedside risk stratification with sufficient accuracy, which was focused on intensive monitoring and therapies for high-risk patients. In addition, the new model was compared with the SRI score and Cleveland Clinic score, with regard to their discrimination, calibration, and clinical usefulness for predicting RRT risk.

## Materials and methods

### Patients

**Figure 1.**
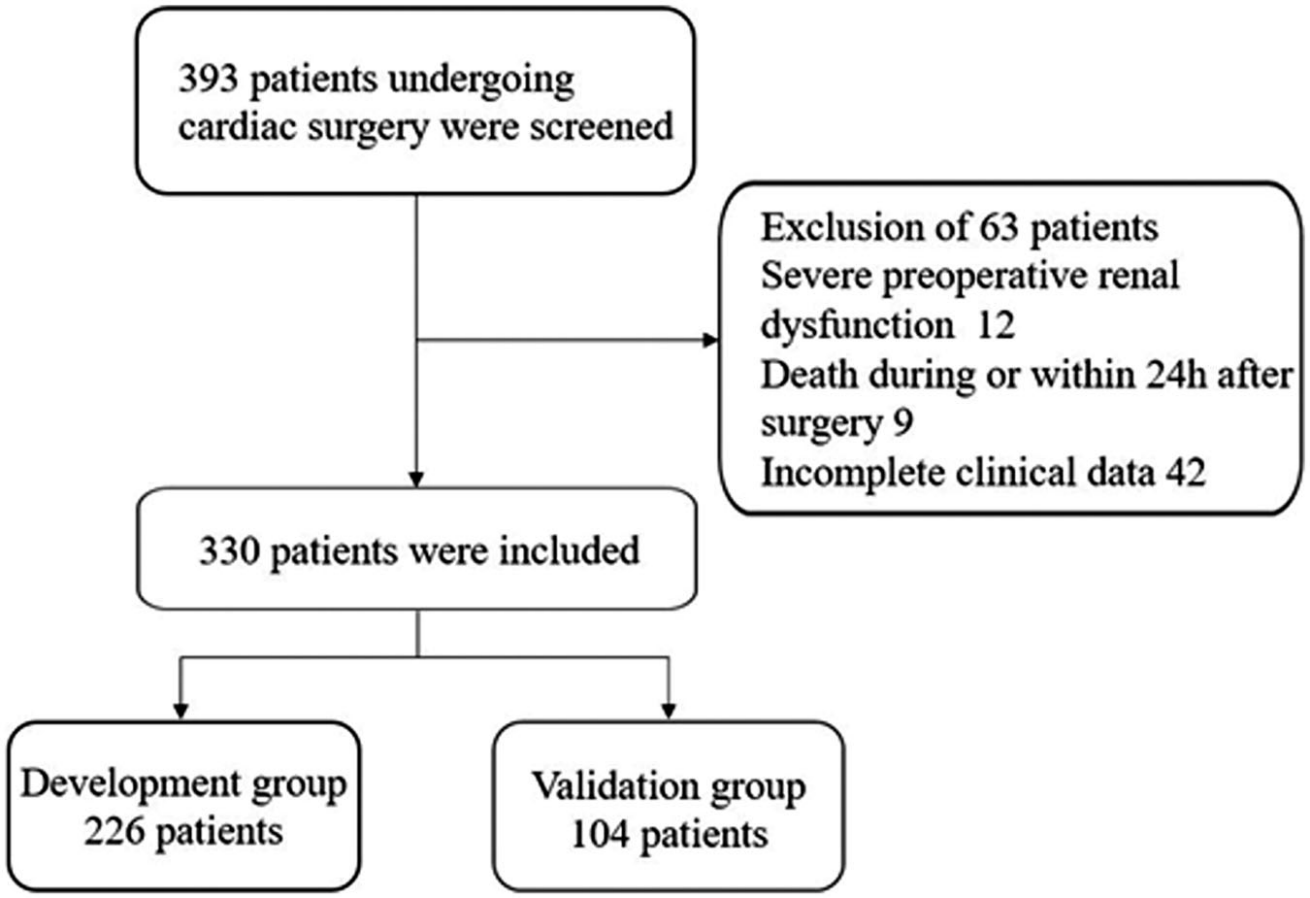
Flowchart of participant selection.

Renal insufficiency patients (estimated glomerular filtration rate [eGFR] based on the Chronic Kidney Disease‐Epidemiology Collaboration formula [12], less than 60 mL/min/1.73 m^2^) older than 18 years old who underwent cardiac surgery at the Affiliated Hospital of Nantong University between January 2011 and January 2019 were collected and retrospectively analyzed. The exclusion criteria were: (1) severe preoperative renal insufficiency (preoperative RRT dependence or eGFR less than 15 mL/min/1.73 m^2^), (2) death during or within 24 h after surgery, (3) incomplete clinical data. For some patients who had more than one cardiac surgery procedure performed during the study period, only the first surgical episode was considered. A total of 393 patients were registered. According to the application of the inclusion and exclusion criteria, 63 patients were excluded. The remaining 330 patients were enrolled in the current study. These patients were randomly assigned to the development group and validation group at a ratio of 7:3. A detailed flow chart of patient selection was shown in Figure 1.

This study was approved by the Ethics Committee of the Affiliated Hospital of Nantong University (approval number: 2020-K096-01) and conducted according to the Declaration of Helsinki. This study was registered in the Chinese Clinical Trial Registry (ChiCTR2100043161).

### Outcomes

The primary endpoint was AKI which required dialysis during the postoperative period. The indications for dialysis based on the clinical judgment by senior nephrologists, included uremia, volume overload, and biochemical abnormalities.

### Data collection

Attempts to improve clinical outcomes of patients with AKI have centered on early diagnosis and customized treatment. Considering that arriving in the intensive care unit (ICU) after surgery is a better time point for intervention, the risk factors of three periods were included in our study: pre-operation, intra-operation, and post-operation immediately (up to 4 h from the ICU admission time). The clinical variables from the electronic medical records of patients were extracted. Demographic characteristics included gender, age, and Body Mass Index (BMI). Preoperative data: diabetes, hypertension, cerebrovascular disease, recent contrast agent exposure (within 7 days before surgery), recent myocardial infarction (occurred within 1 month before surgery), previous cardiac surgery, intra‐aortic balloon pump (IABP), chronic obstructive pulmonary disease, baseline eGFR, emergency surgery, laboratory parameters at admission (hemoglobin, leucocyte, platelet, albumin, alanine aminotransferase, aspartate aminotransferase, blood electrolyte levels, serum creatinine, serum uric acid, serum cystatin C, blood urea nitrogen, B-type natriuretic peptide, high sensitivity C-reactive protein), New York Heart Association (NYHA) functional class was assessed and echocardiography was performed before cardiac surgery, left ventricular ejection fraction, Interventricular septum thickness (IVST) and left ventricular end diastolic diameter (LVEDD) were measured as recommended, the usages of medications (nonsteroidal anti-inflammatory drugs [NSAID], vasoactive drugs, antibiotics, angiotensin‐converting enzyme inhibitors or angiotensin receptor inhibitors [ACEI/ARB], diuretics) before surgery were recorded. Intraoperative data: operative procedure, aortic cross‐clamping time, cardiopulmonary bypass time, IABP, erythrocyte transfusion. Immediate postoperative data: serum creatinine, serum uric acid, serum Cystatin C, blood urea nitrogen, eGFR, medications (NSAID, vasoactive drug, antibiotics, ACEI/ARB, diuretics), IABP.

### Statistical analysis

The Kolmogorov-Smirnov test was used to perform the normality test for continuous variables, and a comparison of the differences between the two groups was performed by using the *t* test, chi-square test, or Mann-Whitney *U* test. The results were presented as mean ± standard deviation ($\bar{\text{x}}\pm \text{SD}$), frequencies (percentages) or median (interquartile range). The least absolute shrinkage and selection operator (LASSO) regression analysis was used for the best predictors of RRT after cardiac surgery. This approach was able to avoid issues of multicollinearity and overfitting, even with a high number of potential predictors and a small sample size. Five‐fold cross‐validation and the 1‐SE rule were performed to control overfitting [13]. All predictors were included in the logistic regression, and a new multivariable regression model was developed. A nomogram was constructed based on the multivariate analysis; the weighted point was calculated by the beta coefficient of each variable in the model. The variable with the highest beta coefficient was scored on a 100 points scale, and the remaining variables were scored according to their individual weighted effect. Finally, the total number of points was calculated [14]. The nomogram was subjected to 1,000 bootstraps resamples for internal validation of the development set. At the same time, the performance of the new model was also analyzed in the verification set and compared with that of the SRI score [10]and Cleveland Clinic score [9]. The area under the receiver operating characteristic curve (AUC) was used to assess discrimination and compared by the DeLong method [15]. A calibration curve was plotted to evaluate the calibration and was accompanied by the Hosmer–Lemeshow test. Finally, a decision curve was carried out to calculate the net benefits of individuals in different threshold probabilities and confirmed the clinical efficacy of the predictive model [16]. All data analysis was performed by SPSS 22.0 software (IBM Corporation, 2013, USA) and R software version 4.0.2 (http://www.r-project.org). The difference was considered to be statistically significant at *p* < 0.05.

## Results

### Characteristics of patients undergoing cardiac surgery in development and validation cohorts

The demographic and baseline clinical data of 330 patients were listed in Table 1; 226 and 104 patients were divided into the development and validation groups, respectively. The rate of RRT in the derivation group was 10.6% (*n* = 24), while the rate in the validation group was 14.4% (*n* = 15). There was no significant difference in baseline characteristics and intraoperative variables between the development and validation groups.

**Table 1.** Baseline characteristics of development and validation group.

Variables	Development group (*n* = 226)	Validation group (*n* = 104)	*P* value
Demographics			
Age (years)	63 ± 10	64 ± 10	0.609
Gender (male)*	133 (58.8)	57 (54.8)	0.490
Body mass index (kg/m^2^)	24.01 ± 3.16	24.41 ± 3.68	0.310
Preoperative			
Hypertension*	110 (48.7)	46 (44.2)	0.453
Diabetes mellitus*	28 (12.4)	18 (17.3)	0.231
Contrast media exposure*	100 (44.2)	39 (37.5)	0.249
Previous cardiac surgery*	7 (3.1)	4 (3.8)	0.725
Recent myocardial infarction*	51 (22.6)	18 (17.3)	0.275
Cerebrovascular disease*	13 (5.8)	6 (5.8)	0.995
Chronic obstructive pulmonary disease*	3 (1.3)	1 (1.0)	0.778
IABP*	14 (6.2)	4 (3.8)	0.383
NYHA classification III or IV*	110 (48.7)	48 (46.2)	0.670
IVST (mm)	10.00 (9.00, 11.00)	10.00 (9.00, 11.00)	0.060
LVEDD (mm)	49.93 ± 8.78	49.66 ± 8.07	0.790
LVEF (%)	59 (55, 64)	58 (53, 64)	0.379
Drugs use*			
Antibiotic	71 (31.4)	44 (42.3)	0.054
ACEI/ARB	74 (32.7)	34 (32.7)	0.993
NSAID	5 (2.2)	0 (0)	0.126
Diuretic	170 (75.2)	79 (76.0)	0.885
Vasoactive drug	8 (3.5)	4 (3.8)	0.890
Hemoglobin (g/L)	127.89 ± 16.70	130.66 ± 19.46	0.211
Leucocyte (×10^9^/L)	5.91 ± 1.62	6.08 ± 1.95	0.424
Platelet (×10^9^/L)	186.78 ± 56.46	198.41 ± 66.23	0.101
Albumin (g/L)	38.38 ± 3.66	38.42 ± 3.45	0.926
Alanine aminotransferase (U/L)	23.50 (15.00, 39.00)	26.00 (17.00, 35.75)	0.587
Aspartate aminotransferase (U/L)	25.00 (21.00, 33.25)	25.00 (21.00, 29.75)	0.561
Natremia (mmol/L)	141.00 (140.00, 143.00)	141.00 (139.00, 143.00)	0.681
Potassium (mmol/L)	4.00 (3.80, 4.30)	4.00 (3.80, 4.20)	0.291
Magnesemia (mmol/L)	0.94 (0.89, 1.00)	0.95 (0.88, 1.00)	0.995
Calcium (mmol/L)	2.23 ± 0.11	2.23 ± 0.11	0.777
Phosphorus (mmol/L)	1.18 ± 0.20	1.22 ± 0.20	0.055
Creatinine (μmol/L)	116.34 ± 19.97	117.31 ± 17.70	0.672
Uric acid (μmol/L)	346.50 (287.50, 414.25)	365.50 (306.25, 444.00)	0.213
Cystatin C (μg/L)	1117.30 ± 321.61	1142.00 ± 302.54	0.509
Blood urea nitrogen (mmol/L)	8.61 ± 2.49	8.31 ± 1.99	0.287
eGFR (ml/min/1.73 m^2^)	52.23 ± 7.42	50.45 ± 8.15	0.051
B-type natriuretic peptide (pg/mL)	200.70 (83.93, 530.63)	207.35 (83.53, 477.20)	0.828
High sensitivity C-reactive protein (mg/L)	3.70 (2.19, 5.65)	3.44 (2.24, 6.65)	0.733
Emergency*	18 (8.0)	9 (8.7)	0.832
Intraoperative			
Operative procedure*			0.432
CABG	64 (28.3)	27 (26.0)	
Valve	153 (67.7)	73 (70.2)	
Valve and CABG	5 (2.2)	4 (3.8)	
Others^#^	4 (1.8)	0 (0)	
Aortic cross‐clamping time (min)	67 (0, 79)	67 (52, 85)	0.385
CPB time (min)	106 (0, 122)	107 (88, 122)	0.417
IABP*	17 (7.5)	7 (6.7)	0.797
Erythrocyte transfusion (U)	3 (3, 3)	3 (3, 3)	0.172
Immediate postoperative^&^			
Creatinine (μmol/L)	153.87 ± 37.83	158.22 ± 40.50	0.343
Uric acid (μmol/L)	371.50 (310.50, 451.25)	392.00 (318.00, 478.50)	0.239
Cystatin C (μg/L)	1304.20 ± 313.04	1349.40 ± 291.42	0.213
Blood urea nitrogen (mmol/L)	11.24 ± 3.51	11.28 ± 3.72	0.914
eGFR (ml/min/1.73 m^2^)	38.67 ± 9.67	36.86 ± 10.02	0.118
Drugs use*			
NSAID	22 (9.7)	3 (2.9)	0.029
Vasoactive drug	221 (97.8)	104 (100)	0.126
Antibiotic	224 (99.1)	104 (100)	0.336
ACEI/ARB	82 (36.3)	40 (38.5)	0.703
Diuretic	225 (99.6)	102 (98.1)	0.188
IABP*	19 (8.4)	11 (10.6)	0.524
Acute kidney injury*	154 (68.1)	77 (74.0)	0.277
Renal replacement therapy*	24 (10.6)	15 (14.4)	0.320
Inhospital mortality*	4 (1.8)	2 (1.9)	0.923

IABP, intra‐aortic balloon pump; NYHA, New York Heart Association; IVST, interventricular septum thickness; LVEDD, left ventricular end-diastolic dimension; LVEF, left ventricular ejection fraction; ACEI/ARB, angiotensin‐converting enzyme inhibitor or angiotensin receptor inhibitors; NSAID, nonsteroidal anti‐inflammatory drugs; eGFR, estimated glomerular filtration rate; CABG, coronary artery bypass grafting; CPB, cardiopulmonary bypass.

* Categorical variables were expressed in frequency (percentage).

# Other surgery includes replacement of the ascending aorta and surgery of atrial septal defects.

& Up to 4 h from the ICU admission time.

The difference was considered to be statistically significant at *p* < 0.05.

### Potential variables used in the model for predicting the risk of RRT

**Figure 2.**
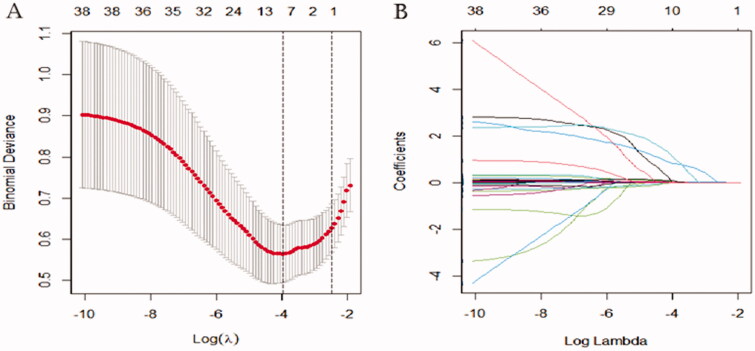
Least absolute shrinkage and selection operator (LASSO) binary logistic regression analysis in prediction of RRT. (A) The optimal parameter (*λ*) of Lasso is selected by the minimum criterion for five times cross-validation. The dotted vertical lines were plotted at the optimal values using the minimum criteria and the one standard error of the minimum criteria (the 1 − SE criteria). Finally, the *λ* value of 0.0191 was selected. (B) The distribution of the lasso coefficient of fifty-five variables. A coefficient profile plot was produced against the log (*λ*) sequence. Predictors were selected based on the minimum criteria, where the best *λ* produced fourteen predictors with non-zero coefficients.

The results of the LASSO regression analysis were presented in Figure 2. A total of 55 potential variables were used in LASSO regression analyses, which were collected from preoperative, intraoperative, and immediate postoperative data. Among of the 55 variables, eight predictors such as postoperative creatinine, aortic cross‐clamping time, emergency, preoperative cystatin C, high sensitivity C-reactive protein, leucocyte, preoperative eGFR, and erythrocyte transfusion were selected to construct a new model for predicting the risk of RRT through logistic regression. In multivariate analysis, postoperative creatinine (*p* < 0.001; odds ratio [*OR*]: 1.026), aortic cross‐clamping time (*p* = 0.004; *OR*: 1.026), emergency (*p* = 0.001; *OR*: 14.779), and preoperative cystatin C (*p* = 0.049; *OR*: 1.001) were considered to be independent risk factors for RRT (Table 2).

**Table 2.** Multivariate logistic regression analyses of variables selected with LASSO.

Variables	OR	95%CI	*P*
Postoperative creatinine level up to 4 h from ICU admission (μmol/L)	1.026	1.013–1.038	<0.001
Aortic cross‐clamping time (min)	1.026	1.008–1.044	0.004
Emergency (yes/no)	14.779	3.206–68.136	0.001
Preoperative cystatin C (μg/L)	1.001	1.000–1.003	0.049
HsCRP (mg/L)	1.094	0.979–1.223	0.113
Leucocyte (×10^9^/L)	1.218	0.964–1.537	0.098
Preoperative eGFR (ml/min/1.73 m^2^)	0.984	0.928–1.043	0.588
Erythrocyte transfusion (U)	1.318	0.738–2.354	0.352

HsCRP, High sensitivity C-reactive protein, eGFR, estimated glomerular filtration rate; OR, odds ratio; CI, confidence interval.

### A nomogram model for prediction of RRT risk

**Figure 3.**
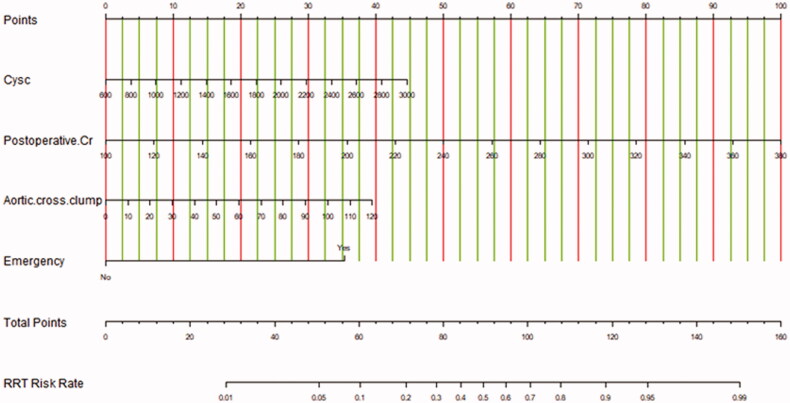
Prediction of RRT in renal insufficiency patients after cardiac surgery by nomogram model. In order to get every factor’s position on the corresponding axis, lines were drawn on the point axis to represent the number of points. Added all points, find the position of the total score to determine the RRT probability of that line in the nomogram. Cys C, preoperative cystatin C (μg/L); Cr, creatinine (μmol/L); RRT, renal replacement therapy.

**Figure 4.**
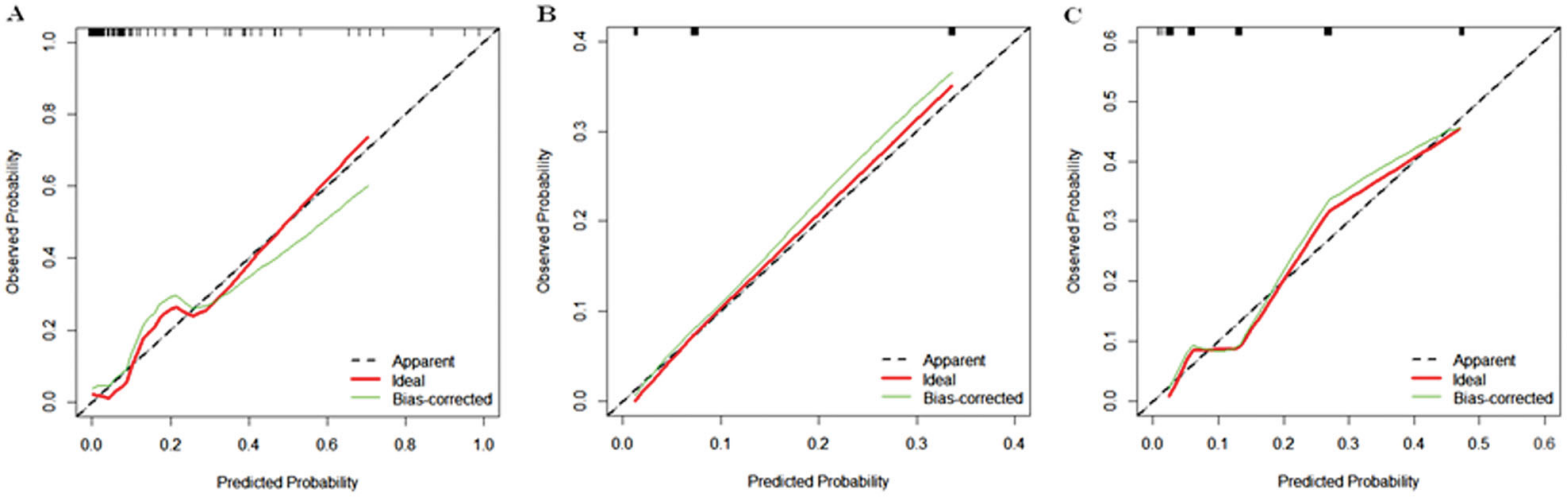
Calibration curves in the validation group for the new model (A), SRI score (B), and Cleveland score (C), respectively. The predicted RRT was plotted on the X-axis, and the actual RRT occurrence was plotted on the Y-axis. A plot along the 45° line would indicate a perfect calibration model in which the predicted RRT is identical to the actual RRT. The dotted line has a close fit to the solid line, which indicated better predictive accuracy of the model.

**Figure 5.**
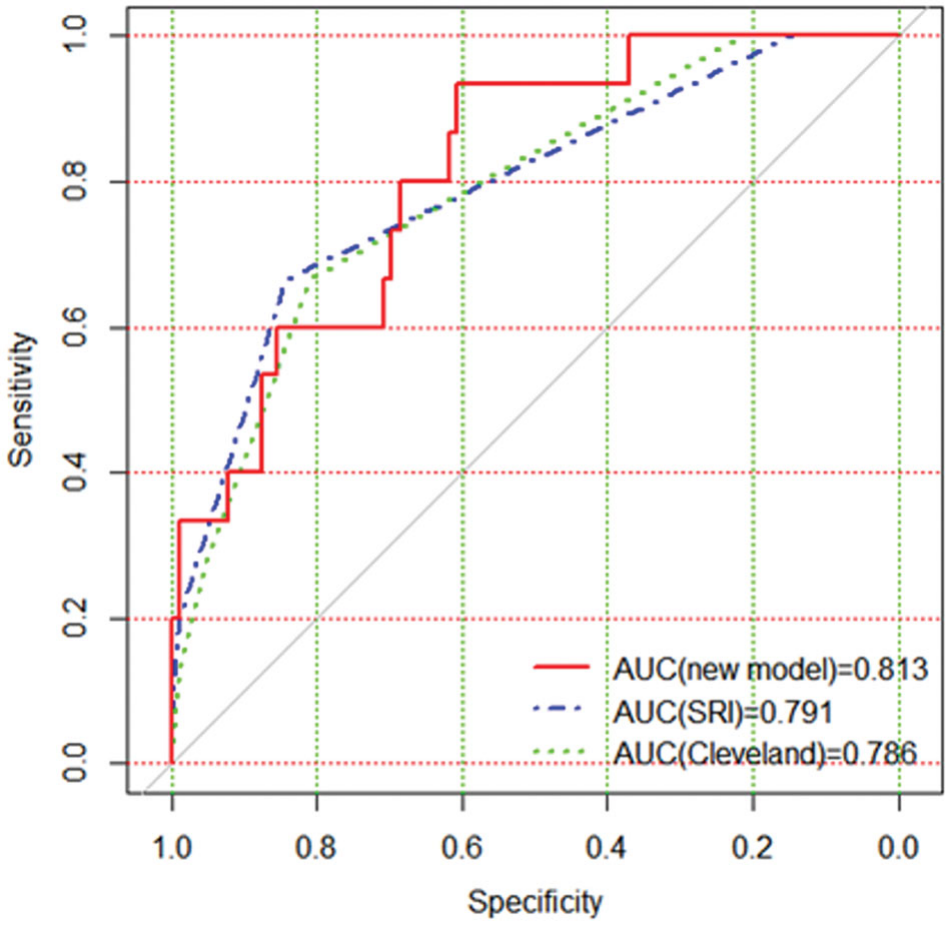
The AUC for models in the validation group. Comparison of AUC among models for RRT in renal inadequacy patients after cardiac surgery. New model AUC: 0.813; SRI score AUC: 0.791; Cleveland Clinic score AUC: 0.786. The new model versus SRI score, *P* = 0.809; new model versus Cleveland score, *P* = 0.746.

A nomogram was also drawn according to the logistic regression results (Figure 3). By creating an intuitive graph of a statistical predictive model, a nomogram showed the numerical probability of each clinical event. For example, the application of this model to renal insufficiency patients undergoing cardiac surgery would show the following results: postoperative creatinine 271 μmol/L, aortic cross‐clamping time 106 min, non-emergency surgery, preoperative cystatin C 800 μg/L, the total score of 100, and the risk of RRT was about 68%. The calibration curves of the new model showed a good agreement (Figure 4), the C-index of the new model was 0.851 (95% CI, 0.779–0.924) in the validation group. Compared with the SRI score and Cleveland Clinic score in the validation group, the AUC for our model, SRI score and Cleveland Clinic score were 0.813, 0.791, and 0.786, respectively. Our new model versus the SRI score or Cleveland Clinic score demonstrated a non-significant difference (*p* = 0.809 and 0.746, respectively, Figure 5).

### Decision curve analysis for the nomogram in the validation group

**Figure 6.**
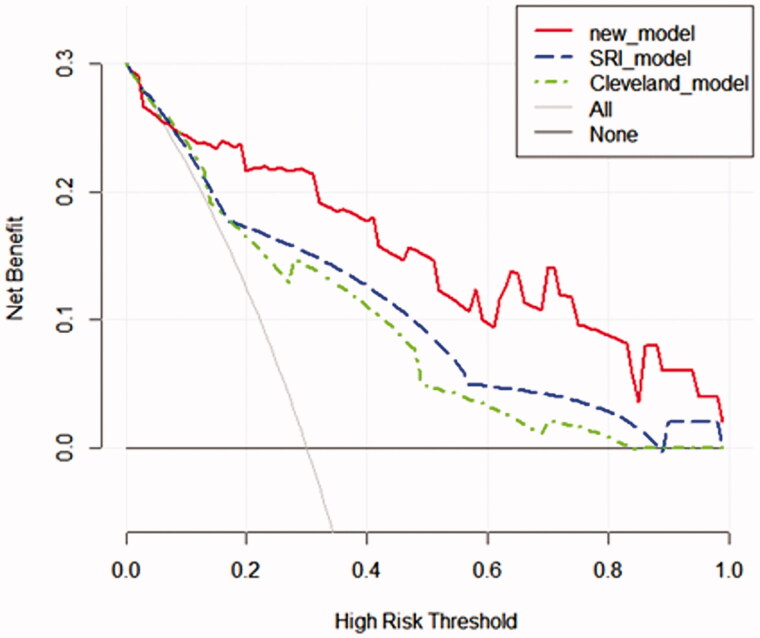
Decision curve analyses for prediction models. The x‐axis shows the threshold probability. The y‐axis shows the net benefit. The black solid lines hypothesized that all patients were RRT positive or negative, respectively. Across the range of decision thresholds, the new model was positive and had a larger net benefit than the SRI and Cleveland scores.

The decision curve analysis displayed that the net benefit of the prediction model was higher within almost all ranges of prediction thresholds, this new model obtained greater clinical net benefits in comparison with the Cleveland Clinic score or SRI score (Figure 6).

## Discussion

AKI is one of the most common serious complications after cardiac surgery [17]. The pathogenesis of postoperative AKI is multifactorial and there are various potential causative mechanisms, such as inflammatory reactions, ischemic‐reperfusion injury, hemolysis, nephrotoxic agents, and oxidative stress [18]. Patients with renal dysfunction before surgery are more likely to experience AKI and are associated with increased mortality, morbidity, and medical costs [19,20]. In recent years, researchers have been committed to the early diagnosis and risk stratification of AKI after cardiac surgery, and thereby providing patients with appropriate individualized prevention and treatment strategies. Unlike previous studies, our study is dedicated to developing and validating a novel predictive tool to identify the risk of RRT in renal insufficiency patients after cardiac surgery. The reason for choosing RRT as the endpoint was based on clinical relevance, and more importantly, considering that AKI requiring hemodialysis after cardiac surgery is probably associated with worse postoperative outcomes [21]. In a study of 13,847 patients, the 1‐year survival rate was reported to be only 10% among patients requiring hemodialysis in the postoperative period [22].

Nomograms have been widely accepted as reliable tools to quantify AKI risk by incorporating and illustrating important factors for AKI [23–25]. In our study, we built a simple and clear nomogram model, the RRT risks were calculated by adding each point according to different parameters, which was more simple and more practical for clinicians compared to the Cleveland Clinic score and SRI score. To date, several clinical risk models for predicting severe AKI after cardiac surgery have been developed, including the SRI score and Cleveland Clinic score, which have been widely validated in patients undergoing cardiac surgery and have been shown to have a better clinical value. However, so far as we know, most existing models have focused on patients undergoing cardiac surgery with normal preoperative renal function. In the general population, the incidence of RRT after cardiac surgery is low and occurs in the later stage in clinical practice, which limits the application of these models. Furthermore, with the development of medical technology, cardiac surgery is booming in patients with renal insufficiency in developing countries, such as China. Lastly, patients with renal insufficiency often have different comorbidities and risk factors for AKI than general patients. Therefore, there is a specific need for an appropriate model to guide clinical management, which is capable of predicting AKI in patients with renal insufficiency undergoing cardiac surgery. As shown in Figure 3, the four top risk factors were included in the new model, including postoperative creatinine, aortic cross‐clamping time, emergency, and preoperative cystatin C. Nomograms have been widely used to quantify the combined contribution of several risk factors and provide a predicted probability of the event of interest. Their prediction applies to an individual instead of situating this individual within a risk group [26–29].

One of the risk factors for RRT is preoperative baseline renal function. The results of our study showed that a preoperative increase in cystatin C was associated with a higher risk of RRT after cardiac surgery. Serum cystatin C has recently been proposed as an alternative marker to serum creatinine for estimating renal clearance, due to its serum levels unaffected by variables other than kidney function [30]. Some equations using serum cystatin C levels to estimate the GFR with or without anthropometric data have recently been reported [31–33]. In addition, Yuan et al. and Saydam et al. reported that serum cystatin C is a reliable biomarker in the early detection and follow-up of AKI after cardiac surgery in a cohort of children and adults, respectively [34,35]. Recently, another study also indicated that a predictive nomogram incorporating cystatin C was helpful to evaluate the possibilities of AKI in patients with traumatic brain injury [36].

Although a remarkable increase in serum creatinine concentration needs a longer time to be detected, it is still a robust variable for predicting the outcome in patients after cardiac surgery [37]. Serum creatinine is conversely and exemplarily associated with poor renal perfusion [38]. Furthermore, it is indicated that a little increase in serum creatinine without evolution to an acutely uremic condition could serve as a predictor of poor prognosis [37]. It appears that microcirculation disturbances leading to local ischemia and reperfusion phenomena determine very early with the initiation of a systemic inflammatory response. For renal insufficiency patients, the early rise in serum creatinine concentration after cardiac surgery may be more significant, at least in our research.

The cardiopulmonary bypass (CPB) time and emergency have been identified as risk factors for AKI after cardiac surgery in previous studies. The CPB provokes a complex systemic inflammatory response, mainly triggered by contact activation of blood by artificial surfaces [39]. Furthermore, the extracorporeal circuit provides nonpulsatile blood flow that may dysregulate the balance between cortical and medullary perfusion in the patient's kidney [40]. Again, aortic cross‐clamping further exacerbates ischemia and induces inflammation [41]. So, it is reasonable that aortic cross‐clamping time is a variable factor that contributes to the development of AKI. CPB time was not included in the model as it was highly correlated with aortic cross‐clamping time [42].

Validation of the nomogram is essential to avoid overfitting the model and determine generalizability [43]. In our study, calibration plots showed optimal agreement between prediction and actual observation, which guaranteed the repeatability and reliability of the established nomogram. Furthermore, compared with that the SRI score and Cleveland Clinic score, the AUC of the new model was the best, and the decision curve analysis displayed that it obtained greater clinical net benefits. The results demonstrated that the new model was simple and clinically useful for predicting RRT immediately postoperatively. The model could be used for clinical risk stratification and to allow for risk stratification for future clinical trials.

The study has several limitations. First, samples were recruited from a single-center, which perhaps introduced bias to the result. In the future, we will apply the model to patients in other centers, hoping to prove the clinical utility of our proposed method in more patients. Second, given its retrospective design, information on some important clinical risk factors of postoperative AKI was not prospectively collected. Therefore, the magnitude of effects of certain risk factors may have been under- or overestimated. Third, the incorporation of novel biomarkers into prediction algorithms may provide additional opportunities to identify patients at high risks, such as heart-type fatty acid-binding protein, midkine, and soluble tumor necrosis factor 1 or 2 [44]. New tools based on novel biomarkers and prospective clinical risk factors need to be developed in future studies with a larger multi-center sample to assist clinicians in identifying cardiac surgery patients at risk of AKI and guide patient management.

Taken together, we developed and validated a powerful predictive model for predicting severe AKI after cardiac surgery in renal insufficiency patients. Furthermore, the new model exhibited greater clinical net benefits than the SRI score and Cleveland Clinic score. It will be used in the perioperative patient management and decision-making about targeted therapies to improve prognosis.
